# *TRPV6* compound heterozygous variants result in impaired placental calcium transport and severe undermineralization and dysplasia of the fetal skeleton

**DOI:** 10.1002/ajmg.a.40484

**Published:** 2018-08-25

**Authors:** Christine P. Burren, Richard Caswell, Bruce Castle, C. Ross Welch, Tom N. Hilliard, Sarah F. Smithson, Sian Ellard

**Affiliations:** ^1^ Department of Paediatric Endocrinology, Bristol Royal Hospital for Children University Hospitals Bristol NHS Foundation Trust Bristol United Kingdom; ^2^ Bristol Medical School Translational Health Sciences University of Bristol Bristol United Kingdom; ^3^ Institute of Biomedical and Clinical Science University of Exeter Exeter United Kingdom; ^4^ Department of Clinical Genetics Royal Devon & Exeter Hospital Exeter United Kingdom; ^5^ Department of Fetomaternal Medicine Derriford Hospital Plymouth United Kingdom; ^6^ Department of Paediatric Respiratory Medicine, Bristol Royal Hospital for Children University Hospitals Bristol NHS Foundation Trust Bristol United Kingdom; ^7^ Department of Clinical Genetics, St Michaels Hospital University Hospitals Bristol NHS Foundation Trust Bristol United Kingdom; ^8^ Department of Molecular Genetics Royal Devon & Exeter Hospital Exeter United Kingdom

**Keywords:** hyperparathyroidism, placental calcium transfer, skeletal demineralization, TRPV6 (transient receptor potential channel 6), skeletal dysplasia

## Abstract

Transient receptor potential vanilloid 6 (*TRPV6*) functions in tetramer form for calcium transport. Until now, *TRPV6* has not been linked with skeletal development disorders. An infant with antenatal onset thoracic insufficiency required significant ventilatory support. Skeletal survey showed generalized marked undermineralization, hypoplastic fractured ribs, metaphyseal fractures, and extensive periosteal reaction along femoral, tibial, and humeral diaphyses. Parathyroid hormone (PTH) elevation (53.4–101 pmol/L) initially suggested PTH signaling disorders. Progressively, biochemical normalization with radiological mineralization suggested recovery from in utero pathophysiology. Genomic testing was undertaken and in silico protein modeling of variants. No abnormalities in antenatal CGH array or UPD14 testing. Postnatal molecular genetic analysis found no causative variants in *CASR*, *GNA11*, *APS21*, or a 336 gene skeletal dysplasia panel investigated by whole exome sequencing. Trio exome analysis identified compound heterozygous *TRPV6* likely pathogenic variants: novel maternally inherited missense variant, c.1978G > C p.(Gly660Arg), and paternally inherited nonsense variant, c.1528C > T p.(Arg510Ter), confirming recessive inheritance. p.(Gly660Arg) generates a large side chain protruding from the C‐terminal hook into the interface with the adjacent TRPV6 subunit. In silico protein modeling suggests steric clashes between interface residues, decreased C‐terminal hook, and TRPV6 tetramer stability. The p.(Gly660Arg) variant is predicted to result in profound loss of TRPV6 activity. This first case of a novel dysplasia features severe but improving perinatal abnormalities. The *TRPV6* compound heterozygous variants appear likely to interfere with fetoplacental calcium transfer crucial for in utero skeletal development. Astute clinical interpretation of evolving perinatal abnormalities remains valuable in complex calcium and bone pathophysiology and informs exome sequencing interpretation.

## 1 INTRODUCTION

Recent advances in molecular genetics have facilitated an expansion and refinement of skeletal dysplasias and metabolic bone conditions diagnosed perinatally (Bonafe et al., 2015).

Fetal skeleton mineralization depends upon fetal plasma calcium sourced through active placental transfer, long attributed to parathyroid hormone (PTH) and PTH‐related peptide activity (Simmonds & Kovacs, 2010). Recently identified additional factors include calbindin (intracellular calcium binding protein), sodium/calcium exchangers (Koo et al., 2012), and TRPV6 (sixth member of the transient receptor potential vanilloid subfamily) (Montell, Birnbaumer, & Flockerzi, 2002).

TRPV6 is relevant to calcium absorption (Fecher‐Trost, Wissenbach, & Weissgerber, 2017). *TRPV6* gene location and expression varies across species; in humans, *TRPV6* is situated on chromosome 7q33‐q34 and is expressed in prostate, placenta, epididymis, and exocrine pancreas (Wissenbach et al., 2001). Research has focused on the potential contribution of *TRPV6* overexpression in prostate and breast cancer. Fewer reports address underexpression: the *trpv6* ^−/−^ knockout mouse has hypofertility due to altered calcium homeostasis in the epididymis and in humans reduced placental expression is associated with preeclampsia (Haché et al., 2011). Until now, *TRPV6* has not been linked with disorders of bone mineralization or skeletal dysplasia. We describe a patient with this phenotype and compound heterozygote *TRPV6* variants occurring in *trans*, illustrating how astute clinical interpretation of evolving perinatal calcium and bone pathophysiology informed the interpretation of trio exome sequencing.

## 2 CASE

A term female infant required significant and prolonged ventilatory support from birth due to severe thoracic insufficiency, accompanied by skeletal and biochemical abnormalities. Management included difficult discussions around survival. Therefore, identifying the underlying diagnosis in this neonate with rare bone disease was invaluable to better inform prognosis.

Antenatal abnormalities were noted early in pregnancy on the 20/40 gestation scan: small chest, unusual rib configuration, short but straight long bones, and no fractures identified. Pregnancy was complicated by severe and reaccumulating polyhydramnios which required three amniotic fluid drainage procedures from 27/40 gestation. There was no preeclampsia. Parents were counseled regarding the severe phenotype of the potentially lethal skeletal dysplasia, with no clear diagnosis.

**Figure 1:**
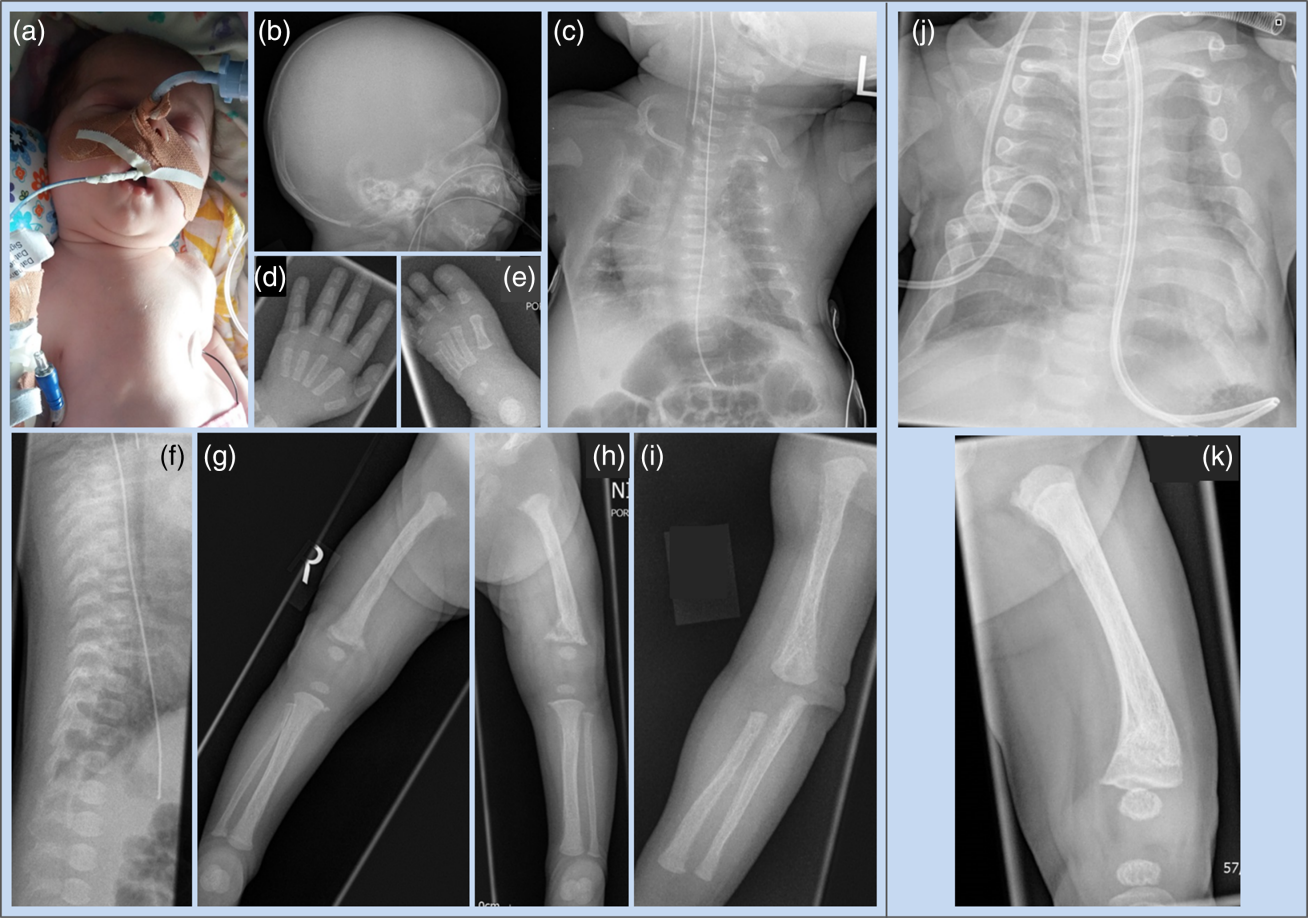
Clinical and radiological findings. (a) The bell‐shaped chest was associated with respiratory distress. (b–i) The skeletal survey at the age of 2 weeks showed generalized undermineralization, short, thin, and fractured ribs, absence of Wormian bones and normal vertebrae. The long bones showed a similar pattern of metaphyseal irregularities with corner fractures and periosteal reaction most obvious along the diaphyses of femora, tibiae and humeri. (j, k) Radiographs of chest and femur at the age of 10 weeks, showed broader, longer ribs, partial resolution of the metaphyseal lesions, and improved bone mineralization [Color figure can be viewed at wileyonlinelibrary.com]

Elective induction of labor at 39^1^/40, emergency cesarean section delivery due to fetal distress, birth weight 3.6 kg, Apgars 3^1^,6^5^,8^10^, and endotracheal intubation and ventilation were required in the delivery room. Clinical features included a marked bell‐shaped chest, otherwise normal proportions, no dysmorphic features and neurologically normal (Figure 1a). Ventilatory requirement persisted; trial of extubation failed Days 4 and 15 and conventional mechanical ventilation was required with maximum pressures of 30 cm H20 and FiO2 0.6. Morphine infusion and paralysis were required for pain attributed to underlying fractures.

The skeletal survey showed widespread undermineralization, short, fractured ribs, metaphyseal irregularities with corner fractures, and periosteal reaction along diaphyses of the long bones (Figure 1b–i).

Early biochemical abnormalities featured markedly elevated PTH 53.4 rising to 101 pmol/L (normal 1.1–6.9 pmol/L), normocalcemia corrected calcium 2.43 mmol/L, normophosphatemia 1.4 mmol/L, normal Alkaline Phosphatase (ALP) 289 IU/L, normal urinary calcium/creatinine ratio 1.05, and vitamin D insufficiency 29 nmol/L. Parental biochemistry was normal.

Neonatal severe hyperparathyroidism (NSHPT) was considered, based on marked PTH elevation and periosteal abnormalities, despite the absence of hypercalcemia; pamidronate single dose Day 16 then daily cinacalcet (calcimimetic) were used preemptively to avoid anticipated severe hypercalcemia (Fisher, Cabrera, & Imel, 2015). PTH normalized, serum calcium did not rise, including after cinacalcet cessation. Transient mild hypocalcemia and hypophosphatemia occurred at weeks 3–4.

At the age of 6 weeks, X‐rays showed improved rib mineralization and width and there was partial resolution of long bone periosteal abnormalities (Figure 1j,k). Ongoing metabolic bone strategy was oral calcium provision as substrate to facilitate bone mineralization and routine vitamin D supplementation (400 IU/day). The ventilatory requirement remained significant but sufficiently improved, with a decision to commence long‐term ventilation through a tracheostomy from 8 weeks.

Throughout 2–4 months of age, skeletal mineralization improved and ventilation stabilized on bi‐level pressures of 16/6 without additional oxygen. Developmental progress was normal. There was a degree of feed volume intolerance attributed to thorax and abdomen size and no other abnormalities identified. Preparation commenced toward transfer and home ventilation, envisaged for approximately 12 months.

Unexpectedly at 4 months, she developed sudden and marked abdominal distension followed by cardiac arrest, profound, and prolonged lactic acidosis. Emergency laparotomy identified volvulus and ischemic bowel. Neurological status postarrest was extremely poor, Magnetic Resonance Imaging (MRI) brain demonstrated global ischemic injury and ultimately care was withdrawn. Limited postmortem confirmed the presence of volvulus, the absence of underlying malrotation, or other gastrointestinal tract configuration abnormalities and mildly enlarged heart with a degree of fiber disarray, although postmortem did not establish a definitive cause of death beyond the presence of volvulus.

## 3 METHODS

Editorial Policies and Ethical Considerations: written consent was obtained from the patient's parents for publication of clinical photographs and clinical information.

Whole exome sequencing was performed with DNA samples from the patient and her unaffected parents using the Agilent SureSelect All Exon v6 system, with sequencing on an Illumina NextSeq 500. Likely causative variants were confirmed by Sanger sequencing.

## 4 RESULTS

Antenatal genetic tests were normal including Array comparative genomic hybridization (aCGH) and investigation for paternal UPD14 (Kagami–Ogata syndrome), undertaken in view of the thoracic appearance.

Postnatally, careful clinical assessment suggested skeletal developmental pathology was the likely primary mechanism resulting in small lungs instead of primary lung pathology (pulmonary hypoplasia). Biochemical abnormalities were not typical of Jeune asphyxiating thoracic dystrophy (in which constrained bone growth would continue postnatally). In contrast, correcting metabolic bone abnormalities could improve bone development aiding rib growth to facilitate lung function.

Initial postnatal genetic testing adopted a targeted single gene approach to explore NSHPT; the initial working diagnosis supported by periosteal changes and marked PTH elevation, although absent hypercalcemia was contradictory. Sanger sequencing of the *CASR* gene did not identify biallelic inactivating variants. We performed whole exome sequencing in the proband to test for variants in the *GNA11* and *AP2S1* genes (less common causes of NSHPT) and also analyzed *GNPTG* as hyperparathyroidism and periosteal changes also occur in I‐cell disease (mucolipidosis Type II). We then applied a broader strategy to explore other causes of bone fragility or skeletal dysplasias using the PanelApp Skeletal Dysplasia Panel Version 1.66, which includes 336 “green” genes (PanelApp skeletal dysplasia panel version 1.66, 2016). No likely causative variants were found through these gene‐focused approaches, excluding a diagnosis of a known form of skeletal dysplasia.

**Figure 2:**
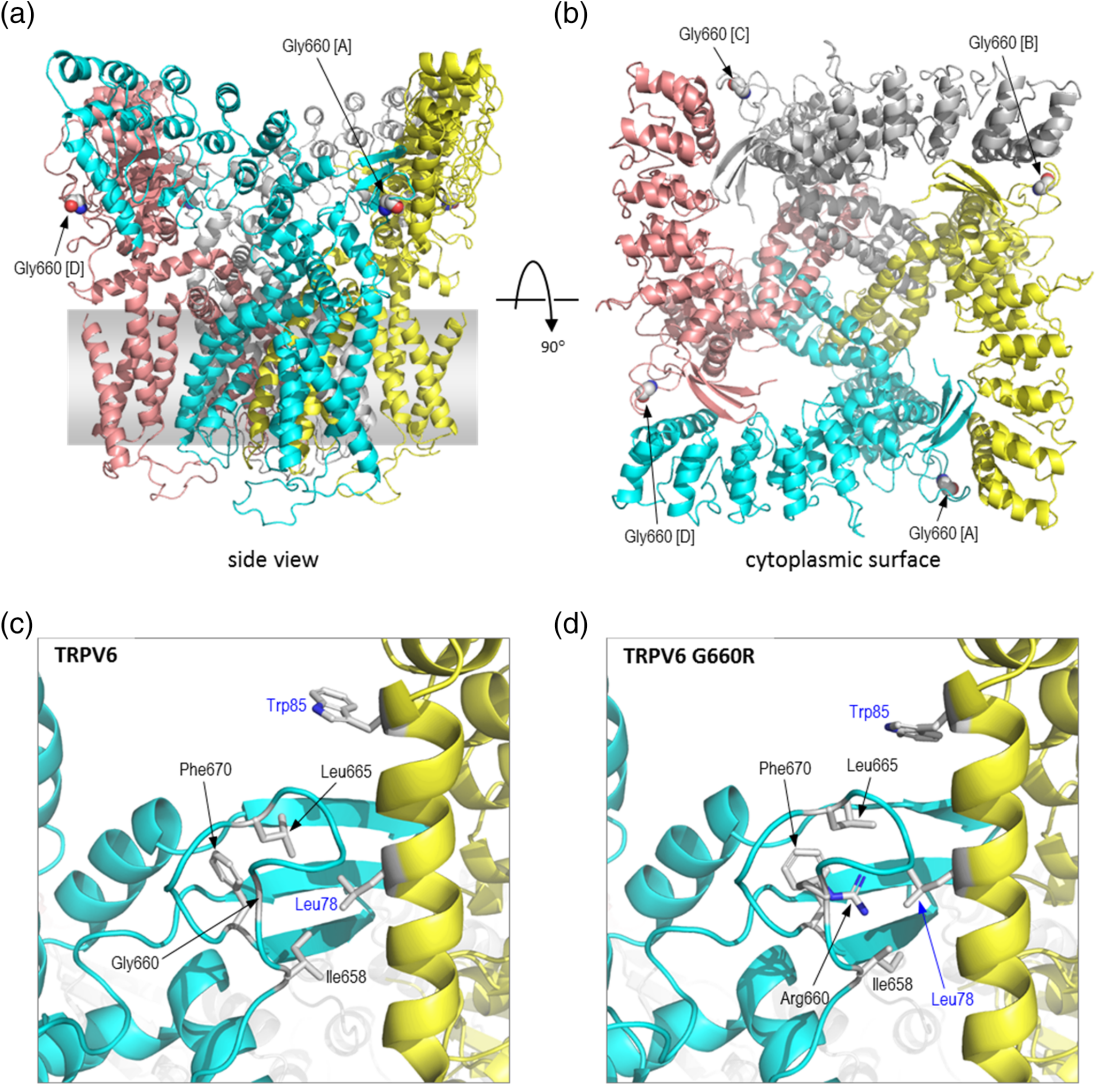
Location of Gly660 and effect of p.(Gly660Arg) substitution. (a) Structure of the human TRPV6 tetramer in open form (PDB id 6bo8); chains A–D are colored cyan, yellow, gray, and pink respectively; atoms of Gly660 are shown in all subunits as spheres colored by atom type (white, carbon; blue, nitrogen; red, oxygen), and are labeled for subunits A and D. The complex is oriented to show view in the plane of the membrane, represented by the gray bar, with the cytoplasmic region at the top. (b) As A, but rotated to show the view from the cytoplasm; the ion channel lies at the center of the tetramer. (c) Detail of the interface between the C‐terminal hook of subunit A (cyan) and the N‐terminal helix of subunit B (yellow); the position of the Gly660 backbone is indicated, and side chains shown in stick format, colored by atom type, for other relevant residues; residues from subunits A and B are labeled in black or blue font, respectively. (d) As A, but showing detail of the p.(Gly660Arg) variant. Variant details: missense is c.1978G > C and nonsense is c.1528C > T. Genomic coordinates Chr7(GRCh37):g.142570162C > G and Chr7(GRCh37):g.142572288G > A) [Color figure can be viewed at wileyonlinelibrary.com]

**Figure 3:**
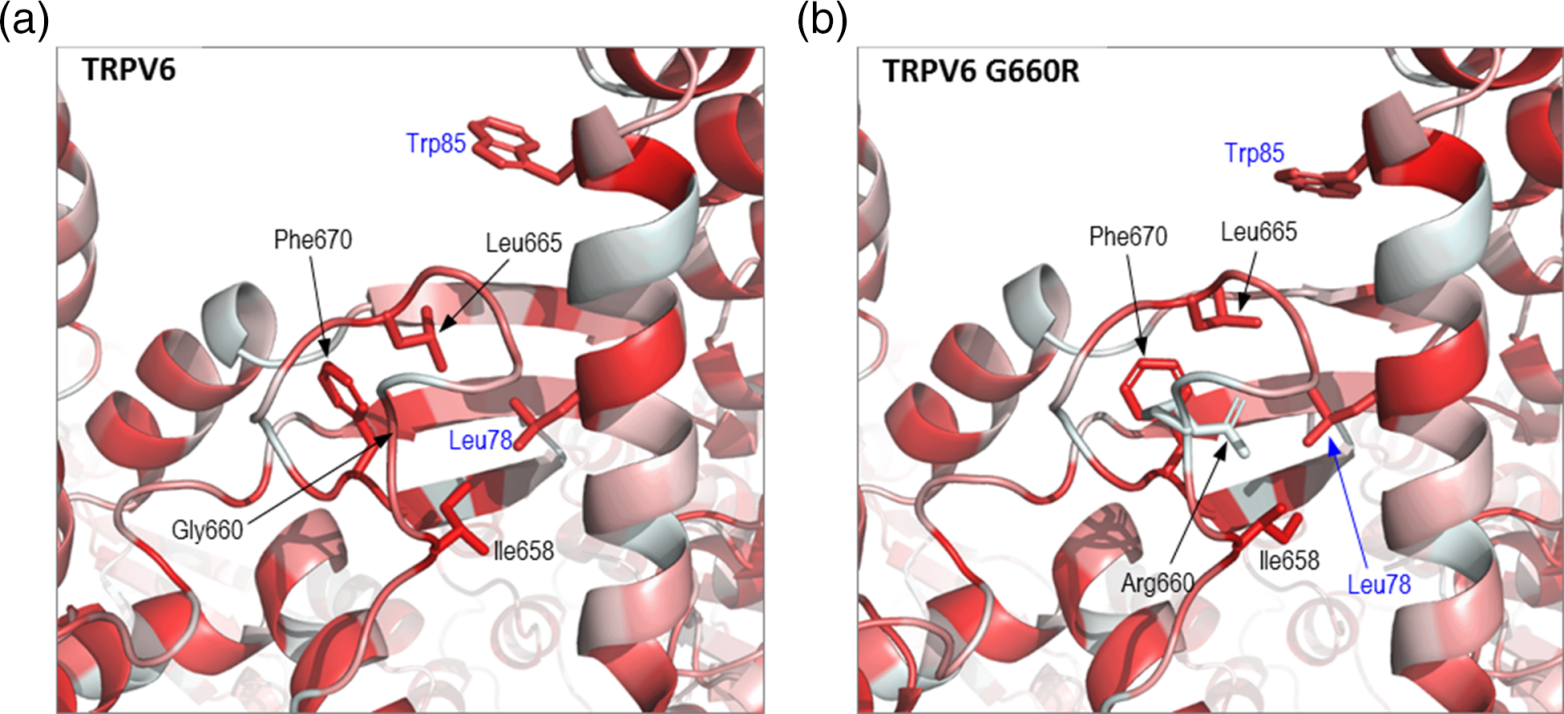
The p.(Gly660Arg) variant disrupts the hydrophobic core of the C‐terminal hook. (a) Detail of the interface between the C‐terminal hook of subunit A and the N‐terminal helix of subunit B, as shown in Figure 2c except that chains are colored by hydrophobicity (red, most hydrophobic; white, most polar). (b) As A, except showing the p.(Gly660Arg) variant [Color figure can be viewed at wileyonlinelibrary.com]

This extensive negative genetic testing, alongside postnatal clinical, biochemical, and radiological changes prompted revision of the intrinsic PTH abnormality hypothesis. Normalized PTH and progressively improved skeletal mineralization suggested the PTH elevation had been reactive to in utero calcium deficiency which progressively resolved in the postnatal environment. The in utero calcium deficiency was not felt to be due to maternal factors (normal biochemistry, health and pregnancy vitamin supplementation). Instead, an intrinsic defect in placental calcium transfer was postulated, prompting direct exploration of the candidate genes *TRPV6, CABP9K*, and *VDR*. We sequenced the unaffected parents and performed a gene‐agnostic trio analysis to identify very rare variants (MAF < 0.0001) compatible with autosomal recessive or de novo inheritance. This identified two genes with possible compound heterozygous single nucleotide variants (SNVs) or indels and one gene with a de novo variant. These included compound heterozygous *TRPV6* variants (NM_018646.5); a novel missense variant, p.(Gly660Arg), and a rare nonsense variant, p.(Arg510Ter) classified according to the ACMG guidelines (Richards et al., 2015) as likely pathogenic (missense: PM2_moderate, PM3_moderate, PP3_supporting, PP4_supporting and nonsense: PVS1_very strong, PM2_moderate). The p.(Arg510Ter) variant lies in exon 11 of 15, and is assumed to induce nonsense‐mediated decay. Glycine 660 lies within the C‐terminal hook region in the intracellular “skirt” of TRPV6 (Saotome, Singh, Yelshanskaya, & Sobolevsky, 2016). The C‐terminal hook packs against the N‐terminal helix of the neighboring subunit, forming an interface at each corner of the skirt (Figure 2a,b). To analyses the effect of the p.(Gly660Arg) substitution, in silico mutagenesis was performed using the Fold X modeling suite (Schymkowitz et al., 2005) to introduce the variant into recently published structures of TRPV6 in its open and closed forms (PDB identifiers 6bo8 and 6boa, respectively, [McGoldrick et al., 2017]). In the predicted structure of the open form of the variant protein, the novel arginine side chain protrudes into the interface between the C‐terminal hook and the N‐terminal helix of the neighboring subunit, resulting in displacement of a number of side chains which contribute both to the interface and the stability of the loop forming the C‐terminal hook (Figure 2c,d). The p.(Gly660Arg) variant also resulted in steric clashes between residues at the interface, and similar observations were made for the closed form of the tetramer (data not shown). The structure of the hook is likely stabilized by a hydrophobic core formed by nonpolar side chains, particularly Leu665, Ile658, and Phe670; the introduction of the large, basic arginine side chain disrupts this environment (Figure 3) and is expected to be energetically unfavorable. Indeed, thermodynamic analysis of the tetramer using FoldX indicated an increase in free energy (i.e., decrease in stability) of the open and closed forms of 73.8 and 61.6 kcal/mol, respectively. Increases in free energy >3 kcal/mol are generally regarded as highly destabilizing to protein structure (Tokuriki & Tawfik, 2009), suggesting that the p.(Gly660Arg) substitution is likely to cause loss of stability at the oligomerization interface and/or reduced formation of the functional tetramer. This may also lead to reduced levels of TRPV6 protein as a result of increased turnover of unstable or misfolded protein. Thus, in combination with the p.(Arg510Ter) allele, the p.(Gly660Arg) variant is predicted to result in a profound loss of TRPV6 activity.

## 5 DISCUSSION

This case adds significantly to understanding of TRPV6 function in humans. We hypothesize that structural/quantitative alterations in TRPV6 in our patient reduced placental calcium transfer and resulted in severely compromised in utero skeletal development and mineralization causing life‐threatening thoracic insufficiency and morbidity from postnatal fractures. Initial differential diagnoses to consider include skeletal dysplasias characterized by poor skeletal mineralization including osteogenesis imperfecta, hypophosphatasia, mucolipidosis Type II, and NSHPT. The unusual clues in this case were in the initial biochemical abnormalities, generally absent in skeletal dysplasias, and their progressive postnatal resolution and bone healing away from the in utero environment.

Finding the precise diagnosis in the patient was fundamental to inform clinical decisions. A hypothesis driven approach to genetic tests contributed first in excluding an unusual presentation of a rare disease (some features suggested NSHPT but severity causing antenatal abnormalities has not been reported) and ongoing hypothesis development complemented a gene‐agnostic trio exome sequence analysis.

Identification of compound heterozygous *TRPV6* variants in this patient coincides with new information on how TRPV6 operates for calcium transfer. TRPV6 crystal structure has been recently described (McGoldrick et al., 2017; Saotome et al., 2016). The gene changes in our patient lie at the tetrameric interface crucial for calcium channel function (McGoldrick et al., 2017) and are predicted to destabilize the protein complex resulting in loss of calcium transport.

Although *trpv6* is expressed in many mouse tissues, including intestine, expression in humans appears restricted to placenta, exocrine pancreas, and some exocrine glands (Fecher‐Trost et al., 2017). Our case showed effects of compromised placental calcium transfer, but did not demonstrate any exocrine pancreatic dysfunction features such as intestinal malabsorption. The absence of preeclampsia does not reinforce the hypothesis of TRPV6 dysfunction in preeclampsia (Haché et al., 2011). Our case showed normal intestinal calcium transfer during early infancy; although oral calcium supplementation was briefly required to compensate for skeletal calcium deficiency, ongoing normal milk calcium content achieved normocalcemia.

The *TRPV* gene family has previously been implicated in skeletal dysplasia: mutations in the *TRPV4* gene are associated with skeletal dysplasia, arthritis‐like, and neurological phenotypes including spondylometaphyseal dysplasia Kozlowski type, brachyolmia Type 3, metatropic dysplasia, spondyloepiphyseal dysplasia Maroteaux type, familial digital arthropathy‐brachydactyly, Charcot–Marie–Tooth disease Type 2C, congenital distal spinal muscular atrophy, and scapuloperoneal spinal muscular atrophy (Krakow et al., 2009; Lamandé et al., 2014; Zimoń et al., 2010). Severe perinatal skeletal dysplasia has not been reported in association with *TRPV6*. Our case has intriguing similarities to severe antenatal‐onset Caffey disease including polyhydramnios, hyperostosis, periosteal reaction, and progressive resolution of bony lesions over time (Schweiger et al., 2003), although we did not observe soft tissue swellings or fever. Antenatal Caffey disease is very rare and although heterozygote *COL1A1* mutations have been identified in some cases (Gensure et al., 2005), genetic heterogeneity seems likely and *TRPV6* may be a candidate worth investigation in unsolved cases.

Combined clinical and genetic expertise achieved diagnostic precision for this case and contributes to understanding of TRPV6 in human disease.

## CONFLICT OF INTEREST

The authors declared that they have no conflict of interest.

## AUTHOR CONTRIBUTIONS

CB: Clinical care of patient including management of calcium homeostasis, recognition of candidate genes for phenotype, main author of this manuscript.

RC: In silico work, analysis of protein structure and consequences of variants in TRPV6, construction of Figure 2, contributor to this manuscript.

BC: Clinical care including antenatal genetic investigation, contributor to this manuscript.

CRW: Clinical care antenatally, approval of final manuscript.

TNH: Clinical care of patient focusing on respiratory management, contributor to this manuscript.

SS: Contribution to phenotypic analysis, interpretation of genetic data and to writing this manuscript.

SE: Scientific design of trio exome sequence analysis, interpretation of genetic data and contributor to this manuscript.
